# Photocurable Vanillin-Derived Hybrid Methacrylate Resins with Tunable Properties for High-Resolution Light-Assisted 3D Printing

**DOI:** 10.1021/acsomega.6c05571

**Published:** 2026-09-07

**Authors:** Nuttapol Risangud, Jittima Mama, Phichanat Iumsriwan, Rattanawadee Srioanchan, Chutidech Thongdeelerd, Tule Sirikitputtisak, Somprasong Thongkham

**Affiliations:** † Petroleum and Petrochemical College, 54776 Chulalongkorn University, Bangkok 10330, Thailand; ‡ 371383 National Nanotechnology Center (NANOTEC), National Science and Technology Development Agency (NSTDA), 111 Thailand Science Park, Phahonyothin Road, Klong Luang, Pathum Thani 12120, Thailand; § Faculty of Science and Industrial Technology, 26686 Prince of Songkla University, Surat Thani Campus, Surat Thani 84000, Thailand

## Abstract

This work reports the development of photocurable hybrid resins based on methacrylated vanillin (MV) and glycidyl methacrylate-functionalized poly­(ε-caprolactone) (PCL-GMA) for high-resolution light-assisted 3D printing. FTIR and ^1^H NMR analyses confirmed successful methacrylation of vanillin and methacrylate end-group incorporation into PCL–GMA, ensuring efficient photopolymerization. Systematic variation of MV/PCL ratios enabled precise control over resin behavior. Cure-depth studies showed that increasing MV content somewhat enhanced photoreactivity. Notably, rheological measurements revealed low viscosities suitable for light-assisted 3D printing, decreasing from 1.55 Pa·s at low MV to 0.10 Pa·s in the MV: PEG system. The 3D-printed specimens exhibited excellent dimensional fidelity, with dimensional errors of 2–3% for cylindrical parts and only 1.17% in height and 1.21% in width for porous diamond lattices. TGA indicated increased thermal stability with higher MV incorporation, while mechanical testing showed significant improvements in modulus and compressive strength across the MV-rich formulations. Taken together, MV/PCL hybrid resins constitute a versatile, low-error, and biocompatible platform for precision liquid crystal display (LCD) based fabrication.

## Graphical abstract

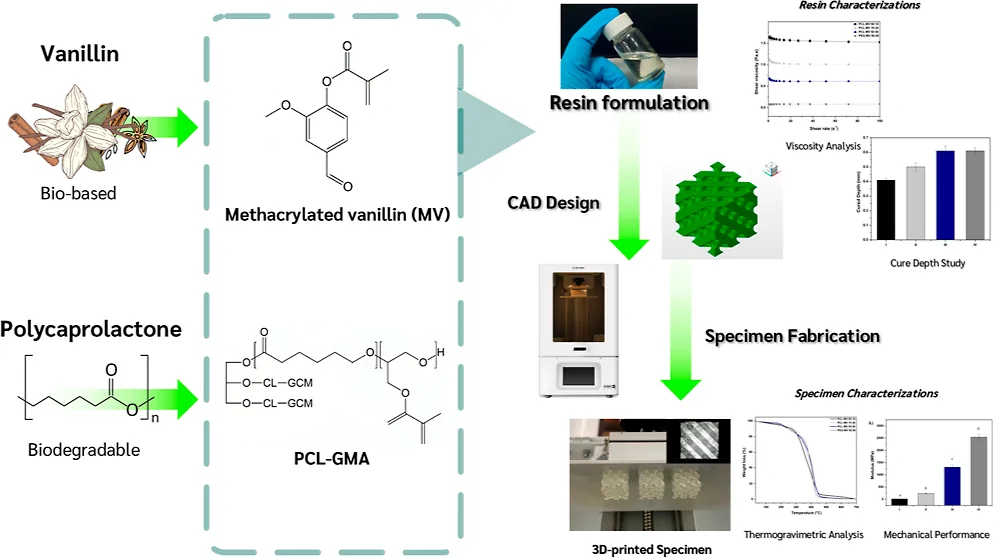

## 1 Introduction

The increasing demand for sustainable polymeric materials has accelerated the development of biobased monomers and oligomers suitable for light-assisted additive manufacturing, particularly digital light processing (DLP), liquid crystal display (LCD), and stereolithography (SLA).^1–4^ Conventional petrochemical-derived acrylates and methacrylates offer excellent photoreactivity but raise environmental concerns and often lack structural diversity. In contrast, biobased feedstocks derived from lignocellulosic biomass, carbohydrates, and plant-derived aromatics provide a versatile platform for designing photocurable networks with reduced environmental impact and enhanced functional tunability.^5–10^ Among these, vanillin—an abundant aromatic aldehyde obtained from lignin depolymerization—has emerged as a promising precursor owing to its intrinsic functional handles that enable straightforward derivatization into (meth)­acrylates, epoxies, esters, and imine-forming monomers. Vanillin-based methacrylate systems have been widely reported to exhibit high photoreactivity, good network rigidity from the aromatic backbone, and potential for reduced volumetric error relative to conventional monomers.^11,12^ These attributes position vanillin-derived monomers and oligomers as an attractive materials platform for developing biobased, photocurable resins that combine sustainability with high performance in light-assisted 3D printing.

Several studies have reported the development of vanillin-based photosensitive resins for light-assisted 3D printing. For example, Liguori et al. developed a series of methacrylated isosorbide and vanillin-based resins to understand how monomer structure influences printability, network rigidity, and recyclability.^13^ Their results showed that isosorbide provides stiffness but can hinder curing when used alone, whereas incorporating vanillin-derived methacrylates improves printing fidelity and network formation. Furthermore, replacing vanillin with a methacrylated Schiff-base monomer introduced dynamic imine linkages, enhancing solvent resistance and enabling both thermal reprocessing and chemical recycling. Notably, the study demonstrated how tuning the balance between rigid biobased aromatics and flexible segments allows control over modulus, glass transition temperature, and recyclability. This highlights the versatility of vanillin-based dynamic networks for next-generation sustainable photopolymers. Wan et al. reported the concept of developing a fully biobased UV-curable resin constructed from methacrylated epoxidized vanillin–ethylenediamine (MEBVE) and methacrylated guaiacol (MG).^14^ Their cured MEBVE–MG networks exhibited high tensile strength (∼63 MPa), a modulus of ∼3 GPa, and excellent thermal stability, while maintaining viscosities suitable for high-precision DLP processing.

Poly­(ε-caprolactone) (PCL)-based photocurable oligomers represent one of the most established soft-segment platforms for light-assisted 3D printing, owing to their biodegradability, cytocompatibility, and favorable chain flexibility.^15–17^ Their aliphatic polyester backbone can be readily functionalized with (meth)­acrylate or glycidyl methacrylate groups, enabling rapid radical cross-linking and seamless integration into DLP workflows.^18,19^ Extensive studies have shown that PCL-derived photopolymers offer broad tunability in mechanical behavior—from compliant elastomers to tougher, semirigid networks—through molecular-weight control, end-group engineering, and crystallinity modulation.^20^ This versatility makes them promising for resorbable tissue-engineering scaffolds, shape-memory devices, and architected lattices used in lightweight, energy-absorbing, or customized load-bearing components. Nevertheless, PCL-based systems often exhibit modest stiffness, limited photoreactivity, and reduced printing resolution when used as standalone matrices, highlighting the need for hybrid strategies that incorporate stiffer or aromatic comonomers to reinforce the network and enhance DLP print performance.

Despite rapid progress in light-assisted additive manufacturing, one persistent challenge limiting the broader adoption of vat-polymerizable materials is polymerization-induced error.^21,22^ Even modest volumetric contraction can lead to interlayer distortion, loss of dimensional fidelity, and failure to reproduce intricate microarchitectures.^23^ Indeed, these issues are repeatedly highlighted across studies of acrylate- and methacrylate-based photopolymers. Error arising from network densification during curing is particularly problematic for biomedical and soft-device applications, where geometric precision is critical. Notably, only a limited number of studies have systematically examined polymerization-induced error and strategies to mitigate it. For instance, Zhang et al. investigated the origins of Error in DLP printing by combining curing kinetics, theoretical modeling, and finite-element analysis (FEA).^24^ They showed that error-induced deformation is closely linked to the evolving degree of conversion (DoC), which dictates both material stiffness and volumetric contraction. Their phase-evolution model—coupling light attenuation, reaction kinetics, network formation, and deformation—successfully predicted layer-by-layer distortion, with experiments validating the simulations. This framework demonstrates that controlling DoC can effectively reduce residual stress and shrinkage, offering valuable guidance for resin formulation and process optimization. Besides, Ling et al. showed that monomer design can also minimize shrinkage.^25^ A resin based on a high-molecular-weight ethoxylated bisphenol A dimethacrylate with minimal TEGDMA achieved the lowest volumetric error with approximately 7% among the tested dental model resins, leading to superior dimensional accuracy. Although progress has been made, effective strategies to suppress error remain limited. This presents an opportunity for polymer chemists to design new photosensitive resins—particularly biobased, biodegradable systems—that inherently minimize shrinkage and improve printing accuracy.

The present work aims to develop a new class of biobased photocurable resins that combine the rigidity and high photoreactivity of vanillin-derived aromatics with the flexibility, biodegradability, and processing advantages of PCL-based soft segments. Although vanillin methacrylates and PCL–GMA oligomers have each been explored independently, their synergistic potential lies in simultaneously reducing polymerization-induced error and tuning mechanical performance. Herein, we introduce a series of hybrid MV/PCL–GMA formulations designed to provide controlled viscosity, enhanced curing behavior, and improved dimensional stability during LCD printing. Through comprehensive evaluation of their photopolymerization characteristics, error behavior, thermal properties, mechanical performance, and cytocompatibility, this study establishes a versatile materials platform that enables high-resolution, low-error, and mechanically tunable biobased resins. By integrating renewable aromatic monomers with biodegradable soft segments, this work demonstrates a practical pathway toward sustainable, high-fidelity photopolymers for light-assisted additive manufacturing.

## 2 Materials and Methods

### 2.1 Materials

Vanillin (VAN, 99%), methacrylic anhydride (MAA, 98%), ε-caprolactone (ε-CL, 97%), glycidyl methacrylate (GMA, 97%), tin­(II) 2-ethylhexanoate (Sn­(Oct)_2_, 92.5%), diphynyl (2,4,6-trimethylbenzoyl) phosphine oxide (TPO), polyethylene glycol dimethacrylate (PEGDMA, *M*_n_ = 750 Da), were purchased from Sigma-Aldrich. Hydroquinone (HQ) was purchased from Fluka. 4-Dimethylaminopyridine (DMAP) from Tokyo Chemical Industry. Sodium bicarbonate (NaHCO_3_), sodium hydroxide (NaOH), magnesium sulfate (MgSO_4_) from Supelco. Dichloromethane (99.8%) from Fisher. Glycerol (Gly) from Chemipan. Hexane and isopropyl alcohol were obtained from Qchemical. All chemicals were used as received without further purification.

### 2.2 Characterizations

^1^H NMR and ^13^C NMR were recorded on a Bruker AVANCE III 400 MHz with CDCl_3_ as the solvent. Monitored by a refractive index detection. FTIR spectra were recorded on a (Thermo Scientific Nicolet iS50) and analyzed with OMNIC software. Particle morphologies were observed by scanning electron microscopy (SEM, Hitachi/S-3400N). Rheological analyses were conducted using a rheometer (Bohin Gemini HR nano 200 nano Rheometer, Malvern Instruments, UK). 3D-printing specimens were fabricated using a liquid crystal display (LCD, Phrozen Sonic XL 4K 2022). Mechanical performance was conducted using a Universal Testing Machine (UTM; Instron model D638, USA). Thermogravimetric analysis was performed using a Mettler Toledo (Hong Kong).

### 2.3 Synthesis of Vanillin Methacrylate

Vanillin methacrylate (MV) was synthesized through esterification of the phenolic hydroxyl group of vanillin with methacrylic anhydride under an inert atmosphere. Vanillin (30 g, 197 mmol) was placed in a 100 mL two-neck round-bottom flask, followed by 4-dimethylaminopyridine (DMAP, 0.503 g, 4.34 mmol), which served as a nucleophilic catalyst. The flask was purged with nitrogen for 30 s while the mixture was stirred. This purge–stir cycle was repeated three times to remove residual oxygen and minimize inhibition or side reactions involving the methacrylate groups.

Subsequently, methacrylic anhydride (33.4 mL, 217 mmol) was added dropwise to the reaction mixture to control the reaction rate and minimize localized overheating. The esterification was then allowed to proceed at 60 °C for 24 h under continuous stirring and a nitrogen atmosphere. After completion, the reaction mixture was cooled to room temperature and diluted with dichloromethane (DCM, 150 mL) to facilitate purification. The organic phase was washed sequentially with 1 M NaHCO_3_, 1 M NaOH, 0.5 M NaOH, and deionized water to remove residual methacrylic anhydride, acidic byproducts, catalyst residues, and other water-soluble impurities. The purified organic layer was dried over anhydrous MgSO_4_, filtered, and concentrated under reduced pressure using a rotary evaporator. The resulting methacrylated vanillin was obtained as a clear, colorless liquid (>75% yield).

### 2.4 Synthesis of Glycidyl-Methacrylate-Functionalized Poly­(ε-Caprolactone)

PCL–GMA was synthesized via ring-opening polymerization (ROP) of ε-caprolactone followed by in situ end-functionalization with glycidyl methacrylate (GMA). Tin­(II) 2-ethylhexanoate (Sn­(Oct)_2_, 0.14 g, 0.345 mmol) and glycerol (2.27 g, 24.7 mmol) were first charged into a two-neck round-bottom flask and stirred under a nitrogen atmosphere for 10 min at 110 °C to remove residual moisture and oxygen. The reaction temperature was then increased to 140 °C, and ε-caprolactone (29.56 mL, 259 mmol) was added. The mixture was stirred for 30 min, or until a clear homogeneous melt was obtained, indicating successful initiation of the ROP process. Subsequently, glycidyl methacrylate (GMA, 12.22 mL, 86 mmol) was added dropwise, and the reaction was allowed to proceed under continuous stirring at 140 °C for 4.5 h. Upon completion, the reaction mixture was cooled to room temperature and dissolved in dichloromethane (DCM, 88 mL). The polymer was precipitated into cold hexane, collected, and dried at room temperature overnight to remove residual solvent. The resulting product was obtained as a viscous liquid (yield 95%).

### 2.5 Photosensitive Resin Formulation

Four photocurable resin formulations were prepared using methacrylated vanillin (MV), PCL–GMA, and PEGDMA at varying weight ratios (Table 1). MV provided aromatic rigidity and reactivity, PCL–GMA contributed flexibility, and PEGDMA served as the difunctional cross-linker. TPO was incorporated at 3 wt % in all formulations. The components were mixed and heated to 40–50 °C until homogeneous, then TPO was added and stirred for 30 min.

**Table 1:** Composition of the Photosensitive Resin Formulations Prepared from Methacrylated Vanillin, PCL–GMA, and PEGDMA at Varying Weight Ratios

formulation	PCL	PEG	MV	TPO (%)	HQ (%)
PCL/MV 90:10	87.12		9.68	3	0.2
PCL/MV 70:30	67.76		29.04		
PCL/MV 50:50	48.40		48.40		
PEG/MV 50:50		48.40	48.40		

To determine the optimal exposure parameters for LCD layer curing, a standard cure test was performed. Approximately three droplets of each resin were placed on a clean glass slide and irradiated for 30 s under the same light source used for 3D printing. After exposure, the unreacted resin was gently wiped off, and the cured film thickness was measured with a digital vernier calliper. The cure depth was calculated and used to determine appropriate exposure times for subsequent layer-by-layer fabrication.

The rheological behavior of each resin formulation was evaluated using a Bohlin Gemini HR Nano 200 Rheometer (Malvern Instruments, UK). A parallel-plate configuration with a 50 μm gap distance was employed. Measurements were performed at room temperature, and steady-shear viscosity was recorded over a shear rate range of 1–100 s^–1^. This analysis provided insights into resin flow behavior and printability under typical light-assisted 3D printing processing conditions.

### 2.6 3D-Printed Specimen Fabrication

The 3D-printed specimens from all formulations were fabricated using a Phrozen Sonic XL 4K (2022) LCD 3D printer. The cylindrical and gyroid models were designed in Autodesk Netfabb and exported as STL files. Printing parameters were defined in Phrozen DS Slicer, with a layer thickness of 100 μm. The exposure time per layer was optimized individually for each formulation based on the initial cure test, ranging from 5 to 7 s. After printing, specimens were washed in isopropyl alcohol (IPA) for 10 min, then sonicated in DI water for 5 min to remove uncured resin. The printed parts were air-dried and subjected to UV postcuring for 30 s. Final dimensions, including specimen diameter and height, were measured using a digital vernier caliper to assess printing accuracy and dimensional fidelity.

### 2.7 3D-Printed Specimen Characterization

#### 2.7.1 Error Measurement

Dimensional accuracy was evaluated using cylindrical specimens fabricated by LCD 3D printing. The corresponding CAD model had a designed height of 3.0 mm and diameter of 8.0 mm. Ten specimens were prepared for each formulation (*n* = 10). The initial height and diameter of each printed specimen were measured using a digital vernier caliper. The specimens were then air-dried for 30 min and subsequently UV-postcured for 30 min. After postcuring, the specimen dimensions were remeasured, and the percentage error was calculated by comparing the postcured dimensions with those of the corresponding CAD model. Error (%) was determined using the following equation

$$\%\;error=\frac{\left(D_{before}-D_{after}\right)}{D_{before}}\times 100$$

where *D*_before_ and *D*_after_ represent the specimen dimensions (height or diameter) before and after postcuring, respectively.

#### 2.7.2 Thermal Characteristics

The thermal stability of the 3D-printed specimens was evaluated using thermogravimetric analysis (TGA). Measurements were performed on a Mettler Toledo TGA system (Hong Kong). Approximately 1.50 mg of each UV-cured sample was placed in an alumina pan and heated from room temperature to 600 °C under a nitrogen flow of 60 mL min^–1^. A constant heating rate of 30 °C min^–1^ was employed. The resulting thermograms were used to determine key degradation parameters, including the onset decomposition temperature and the temperature corresponding to 5% and 50% weight loss.

#### 2.7.3 Mechanical Performance

The mechanical properties of the UV-cured 3D-printed specimens were assessed using a universal testing machine (UTM, Instron model D638, USA) equipped with a 50 kN load cell. Cylindrical samples printed as described in Section 2.5 were used for all tests. compression) tests were performed at a constant crosshead speed of 1 mm min^–1^, following standard testing procedures. The force–displacement data were recorded and converted to engineering stress–strain curves.

#### 2.7.4 Morphological Evaluation

The surface morphology of the UV-cured 3D-printed specimens was examined using a scanning electron microscope (SEM, Hitachi S-3400N). Samples were sputter-coated with a thin layer of gold for 90 s to ensure adequate electrical conductivity. SEM observations were conducted at an accelerating voltage of 15 kV and a working distance of 5 mm.

### 2.8 Statistical Analysis

All tests were conducted using at least three specimens (*n* ≥ 3), and the data are presented as mean ± standard deviation (SD). Statistical analyses were performed using one-way ANOVA followed by Duncan’s multiple range test to compare the curing depth, dimensional errors, and mechanical properties among the resin formulations. All analyses were conducted using IBM SPSS Statistics, version 29.0.2 (IBM Corp., Armonk, NY, USA). Differences were considered statistically significant at *p* < 0.05.

## 3 Results and Discussion

### 3.1 Synthesis and Chemical Characterization of Photopolymers

The chemical structure of methacrylated vanillin (MV) was verified by FTIR and ^1^H NMR spectroscopy. In the FTIR spectra (Figure 1A), pristine vanillin exhibited characteristic absorption bands at 3164 cm^–1^ (O–H stretching of the phenolic group), 1661 cm^–1^ (C=O stretching), 1585 cm^–1^ (aromatic C=C stretching), 1263 cm^–1^ (C–O stretching), and 731 cm^–1^ (C–H bending). Methacrylic anhydride displayed distinct features at 1033 cm^–1^ (C–O stretching), 1636 cm^–1^ (C=C stretching), 1720–1779 cm^–1^ (C=O stretching), and 2931–2987 cm^–1^ (C–H stretching). After esterification, the FTIR spectrum of MV showed combined signatures of both components. The disappearance of the broad O–H band at 3164 cm^–1^ confirmed consumption of the phenolic hydroxyl group, while the emergence of strong C=O stretching at 1720–1779 cm^–1^ and C=C stretching at 1599–1636 cm^–1^ verified the successful introduction of the methacrylate group. Retention of vanillin-derived C–O and C–H bands further confirmed selective modification at the hydroxyl site.

**Figure 1:**
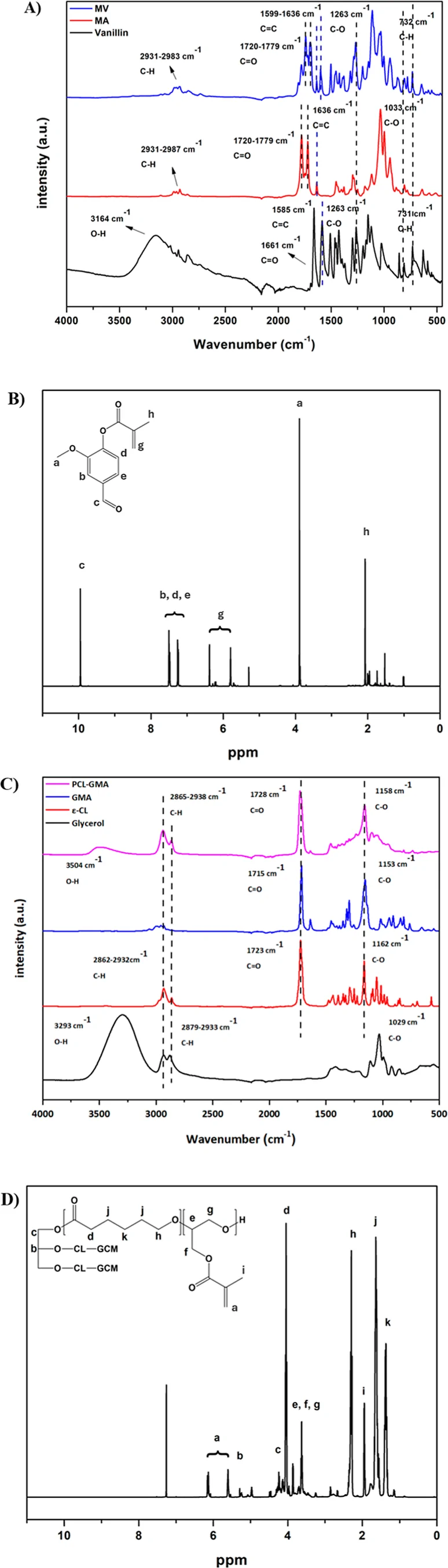
FTIR and ^1^H NMR confirmation of monomer and macromer synthesis. (A,B) Methacrylated vanillin shows the disappearance of the phenolic O–H band and characteristic methacrylate vinyl signals. (C,D) Glycidyl-methacrylate-functionalized poly­(ε-caprolactone) displaying ester and vinyl absorptions and corresponding NMR resonances consistent with methacrylate-terminated PCL.

Besides, the ^1^H NMR spectrum of MV (Figure 1B) further supported the presence of the methacrylate ester. A resonance at 2.3 ppm corresponded to the methacrylate −CH_3_ group, while the methoxy protons of vanillin appeared at 3.9 ppm. The vinyl protons of the methacrylate moiety were clearly observed at 5.8–6.4 ppm, confirming successful esterification. Aromatic protons from the vanillin ring appeared at 7.2–7.5 ppm, and a small peak at about 9.9 ppm corresponded to the aldehyde proton.

The synthesis of glycidyl-methacrylate-functionalized poly­(ε-caprolactone) (PCL–GMA) was likewise confirmed by FTIR and ^1^H NMR spectroscopy, following the synthetic protocol established in our previous work.^26^ FTIR spectra of the precursor monomers, including glycerol, ε-caprolactone, and glycidyl methacrylate (GMA), were compared with the resulting PCL–GMA macromer (Figure 1C). Glycerol exhibited characteristic bands at 3293 cm^–1^ (O–H stretching), 2879–2933 cm^–1^ (C–H stretching), and 1029 cm^–1^ (C–O bending). ε-Caprolactone displayed absorption peaks at 1723 cm^–1^ (C=O stretching), 1162 cm^–1^ (C–O bending), and 2862–2932 cm^–1^ (C–H stretching), while GMA showed prominent bands at 1715 cm^–1^ (C=O stretching) and 1153 cm^–1^ (C–O bending). The FTIR spectrum of PCL–GMA displayed combined features characteristic of a methacrylate-terminated polyester, including strong ester C=O stretching at 1728 cm^–1^, C–O stretching at 1158 cm^–1^, and C–H stretching at 2865–2938 cm^–1^, all consistent with polycaprolactone chain formation. Notably, the emergence of a broad O–H band at 3504 cm^–1^ indicated the presence of a small fraction of terminal hydroxyl groups. In contrast, the shifts and sharpening of carbonyl and ether bands confirmed successful ring-opening polymerization and end-functionalization with GMA.

The ^1^H NMR spectrum of PCL–GMA (Figure 1D) further validated the incorporation of methacrylate end groups and the formation of the expected polyester backbone. Vinyl protons of the methacrylate moiety appeared at 5.61–6.16 ppm, confirming the presence of terminal C=CH_2_ groups. Methylene protons adjacent to the ester linkage resonated at 5.29 ppm, while characteristic −OCH_2_– units of the caprolactone repeat appeared at 4.23 ppm. A resonance at 4.06 ppm was attributed to −HOCH_2_– end groups, indicating the presence of a minor population of hydroxyl-terminated chains. Such hydroxyl residues are typical of polycaprolactone architectures and arise from incomplete end-capping or initiator-derived chain ends; however, their occurrence is minimal and does not interfere with subsequent photopolymerization. Additional multiplets at 3.86–3.63 ppm corresponded to −OCH– and −OCH_2_– groups along the backbone. The aliphatic region exhibited well-defined signals at 2.30 ppm (−COCH_3_), 1.95 ppm (vinyl–C­(CH_3_)), 1.63 ppm (internal −CH_2_−), and 1.38 ppm (terminal/aliphatic −CH_2_−), consistent with the expected chemical environment of the PCL repeating structure.

### 3.2 Photosensitive Resin Characterization

Four photocurable resin formulations were prepared using PCL–GMA, methacrylated vanillin (MV), and PEGDMA, as summarized in Table 1. The formulation design enabled systematic investigation of the contributions of soft, flexible segments (PCL–GMA or PEGDMA) and rigid aromatic segments (MV) to the photopolymerization behavior and printability of the resins. Increasing the MV fraction was expected to enhance the cross-linking density owing to its high vinyl functionality and aromatic rigidity, whereas higher PCL content was anticipated to lower viscosity and impart flexibility to the resulting polymer networks. All formulations contained 3 wt % TPO as the photoinitiator and 0.2 wt % hydroquinone (HQ) as an inhibitor to suppress premature polymerization during storage.

The curing behavior of the resins was evaluated by measuring the cured depth after 30 s of irradiation (Figure 2A). The curing depth increased significantly with increasing MV content (*p* < 0.05). The PCL/MV 90:10 formulation exhibited the lowest cured depth (0.41 ± 0.01 mm), followed by PCL/MV 70:30 (0.50 ± 0.02 mm), while PCL/MV 50:50 (0.61 ± 0.04 mm) and PEG/MV 50:50 (0.61 ± 0.03 mm) showed the highest values and were not significantly different from each other. This trend reflects the higher concentration of reactive double bonds and improved radical propagation in MV-rich systems. The comparable curing depths of the PCL/MV 50:50 and PEG/MV 50:50 formulations suggest that the total methacrylate concentration, rather than the backbone chemistry, was the primary factor governing curing efficiency. These findings demonstrate that MV incorporation markedly promotes photopolymerization, potentially enabling shorter exposure times during LCD printing.

**Figure 2:**
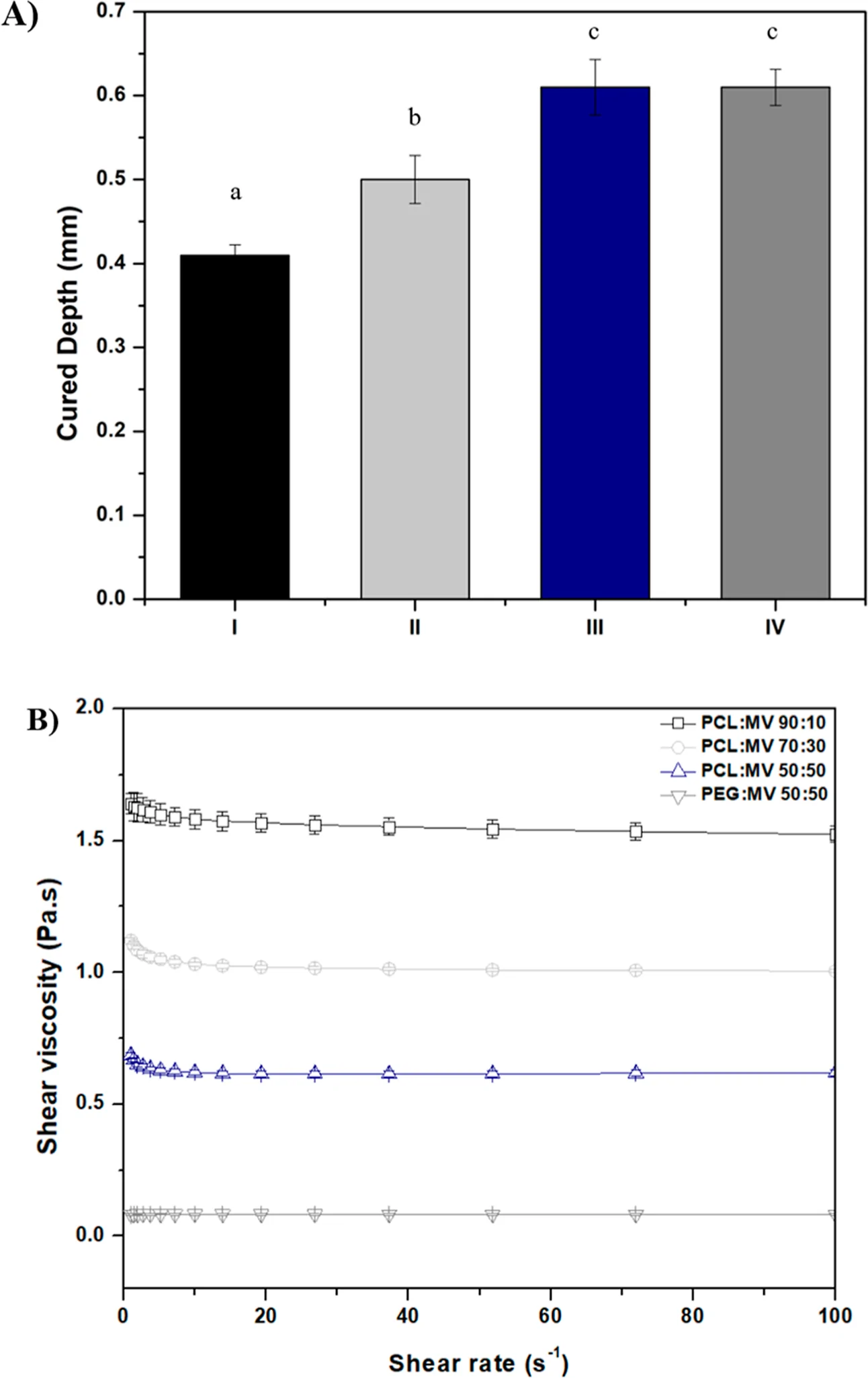
(A) Cured depth of the photocurable resin formulations after 30 s UV irradiation (*n* = 3): (I) PCL/MV 90:10, (II) PCL/MV 70:30, (III) PCL/MV 50:50, and (IV) PEG/MV 50:50. Data are presented as mean ± standard deviation. Different letters above the bars indicate statistically significant differences among formulations (*p* < 0.05), as determined by one-way ANOVA followed by Duncan’s multiple range test. Bars sharing the same letter are not significantly different. (B) Rheological behavior of the resins (*n* = 3), demonstrating systematically reduced viscosity with increasing MV fraction.

Rheological behavior of the resin formulations is shown in Figure 2B. All resins displayed low viscosities suitable for light-assisted 3D printing and exhibited predictable trends based on their component ratios. Viscosity decreased systematically with increasing MV content, attributable to the lower molecular weight and reduced chain entanglement imparted by the monomer-rich formulations. The PCL/MV 90:10 resin exhibited the highest viscosity (about 1.55 Pa·s at 1 s^–1^) due to its elevated fraction of high-molecular-weight PCL–GMA. Increasing the MV content to 30 wt % reduced the viscosity to approximately 1.05 Pa·s, while the 50:50 composition further reduced it to approximately 0.65 Pa·s. The PEG/MV 50:50 formulation demonstrated the lowest viscosity (0.10 Pa·s), reflecting the inherently low viscosity of PEGDMA and its limited contribution to network rigidity. Indeed, all formulations displayed mild shear-thinning behavior at low shear rates, transitioning to a near-Newtonian plateau between 10 and 100 s^–1^. This rheological profile is advantageous for LCD printing, as consistent viscosity under shear supports uniform recoating, while slight shear-thinning enhances resin flow in confined geometries.

### 3.3 3D-Printed Specimen Characterization

#### 3.3.1 Error Measurement

The dimensional stability of the printed cylindrical specimens was assessed before and after postcuring (Table 2). The CAD model and corresponding printed cylindrical specimen used for dimensional error measurements are shown in Figure 3A,B, respectively. Although all formulations showed relatively low dimensional error, clear composition-dependent trends were observed. The PCL/MV 90:10 resin showed a moderate deviation from the CAD model (+2.33% in height and −0.25% in diameter), consistent with its lower methacrylate content and correspondingly reduced cross-link density. Increasing the MV fraction to 30 wt % slightly reduced the height error to +2.00%, while diameter shrinkage increased to −0.88%. In contrast, the PCL/MV 50:50 formulation exhibited the highest height deviation (+3.33%) but comparatively low diameter error (−0.38%). The PEG/MV 50:50 resin showed similar behavior (+3.00% height and −0.90% diameter), reflecting the greater flexibility of the PEG-based network. Overall, these results indicate that methacrylated vanillin content influences dimensional stability through its effect on network structure, with aromatic MV-containing PCL systems maintaining good dimensional fidelity following postcuring.

**Table 2:** Dimensional Stability of 3D-Printed Cylindrical Specimens Before and After Post-Curing^b^

	CAD (mm)		green specimen (mm)		postcured specimen (mm)		% error[Table-fn t2fn1]	
formulation	height	diameter	height	diameter	height	diameter	height	diameter
PCL/MV 90:10	3	8	3.12 ± 0.02	8.05 ± 0.02	3.07 ± 0.03	7.95 ± 0.02	+2.33^b,c^	–0.63^b,c^
PCL/MV 70:30			3.09 ± 0.03	8.07 ± 0.03	3.06 ± 0.01	7.99 ± 0.43	+2.00^c^	–0.13^a^
PCL/MV 50:50			3.11 ± 0.01	8.03 ± 0.02	3.10 ± 0.02	7.97 ± 0.01	+3.33^a^	–0.26^b,c^
PEG/MV 50:50			3.12 ± 0.02	8.01 ± 0.01	3.09 ± 0.02	7.93 ± 0.03	+3.00^a,b^	–0.88^c^

a­( 1) Error relative to the postcured specimen. (2) A “–” sign indicates the printed sample is smaller than the CAD model, while “+” indicates it is larger, and (3) Different letters (a–c) indicate statistically significant differences between formulations (*p* < 0.05), as determined by one-way ANOVA followed by Duncan’s multiple range test. Formulations sharing the same letter are not significantly different.

b Increasing methacrylated vanillin content in PCL-based resins reduced shrinkage (*n* = 10).

**Figure 3:**
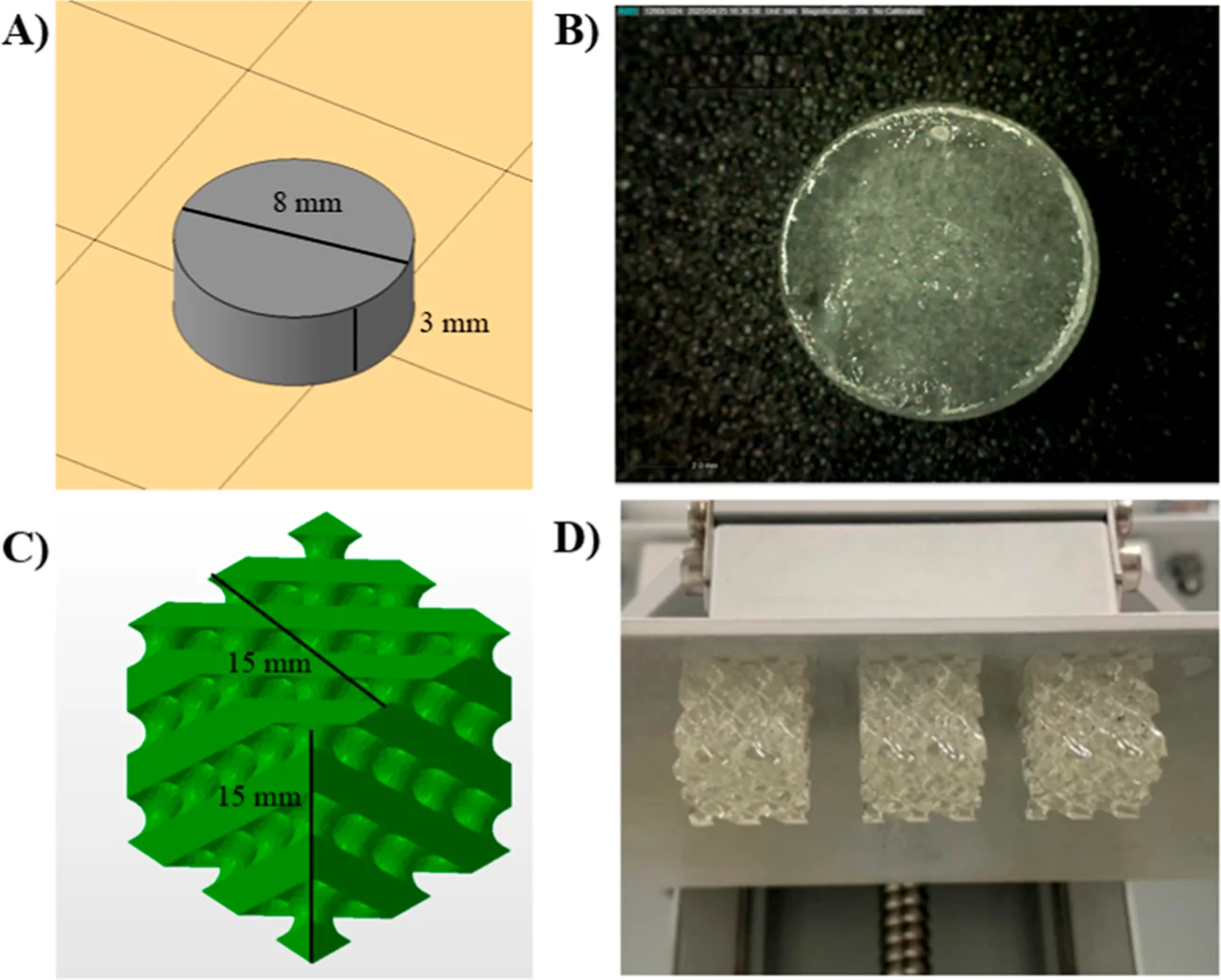
CAD models and corresponding printed specimens: (A) cylindrical model with a diameter of 8 mm and height of 3 mm, (B) printed cylindrical specimen, (C) porous diamond lattice model with overall dimensions of 15 × 15 × 15 mm^3^, and (D) printed porous diamond lattice specimens fabricated using the PCL/MV 50:50 formulation.

To further evaluate dimensional fidelity in more complex geometries, a porous diamond lattice was fabricated using the PCL/MV 50:50 formulation, which previously exhibited the lowest error among the cylindrical models. The lattice structure was designed in Autodesk Netfabb (Figure 3C). The printed constructs showed excellent shape retention and uniform pore distribution, indicating consistent curing throughout the network (Figure 3D). Postcuring dimensional analysis revealed minimal error, averaging 1.17% in height and 1.21% in width—values notably lower than those observed for the porous diamond lattice specimens. The reduced error is attributed to the open-cell geometry, which allows the dissipation of internal stresses generated during postcuring to be more effective than in solid parts. In addition, the high methacrylate content and aromatic rigidity of the PCL/MV 50:50 formulation facilitate rapid network formation and restrict volumetric relaxation under UV postcuring. Overall, the results confirm that the PCL/MV 50:50 resin provides excellent dimensional stability, even in complex lattice architectures, highlighting its suitability for fabricating lightweight, high-precision porous structures via light-assisted 3D printing.

#### 3.3.2 Thermal Characteristics

Thermogravimetric analysis (TGA) was performed to evaluate the thermal stability of the photocured networks (Figure 4). All formulations exhibited a slight initial mass loss near 120 °C, attributed to residual unreacted resin components or low-molecular-weight fragments not fully incorporated into the cross-linked network. A second major degradation stage occurred around 200 °C, corresponding to the thermal decomposition of the cross-linked polymer matrix. Complete combustion of the networks was observed at approximately 441 °C. The temperature at 50% weight loss (*T*_50_) provides vital information for comparing thermal stability. The PCL/MV 90:10, 70:30, and 50:50 formulations displayed *T*_50_ values of 363 °C, 390 °C, and 390 °C, respectively. These results indicate that the main improvement in thermal stability occurred as the MV content increased from 10% to 30%, whereas a further increase to 50% produced no additional increase in *T*_50_. The PEG: MV 50:50 formulation exhibited a *T*_50_ of 391 °C, comparable to that of the PCL-based 50:50 resin, but with slightly lower overall stability. This was likely associated with the more flexible PEGDMA backbone, which introduces fewer thermally robust segments than PCL.

**Figure 4:**
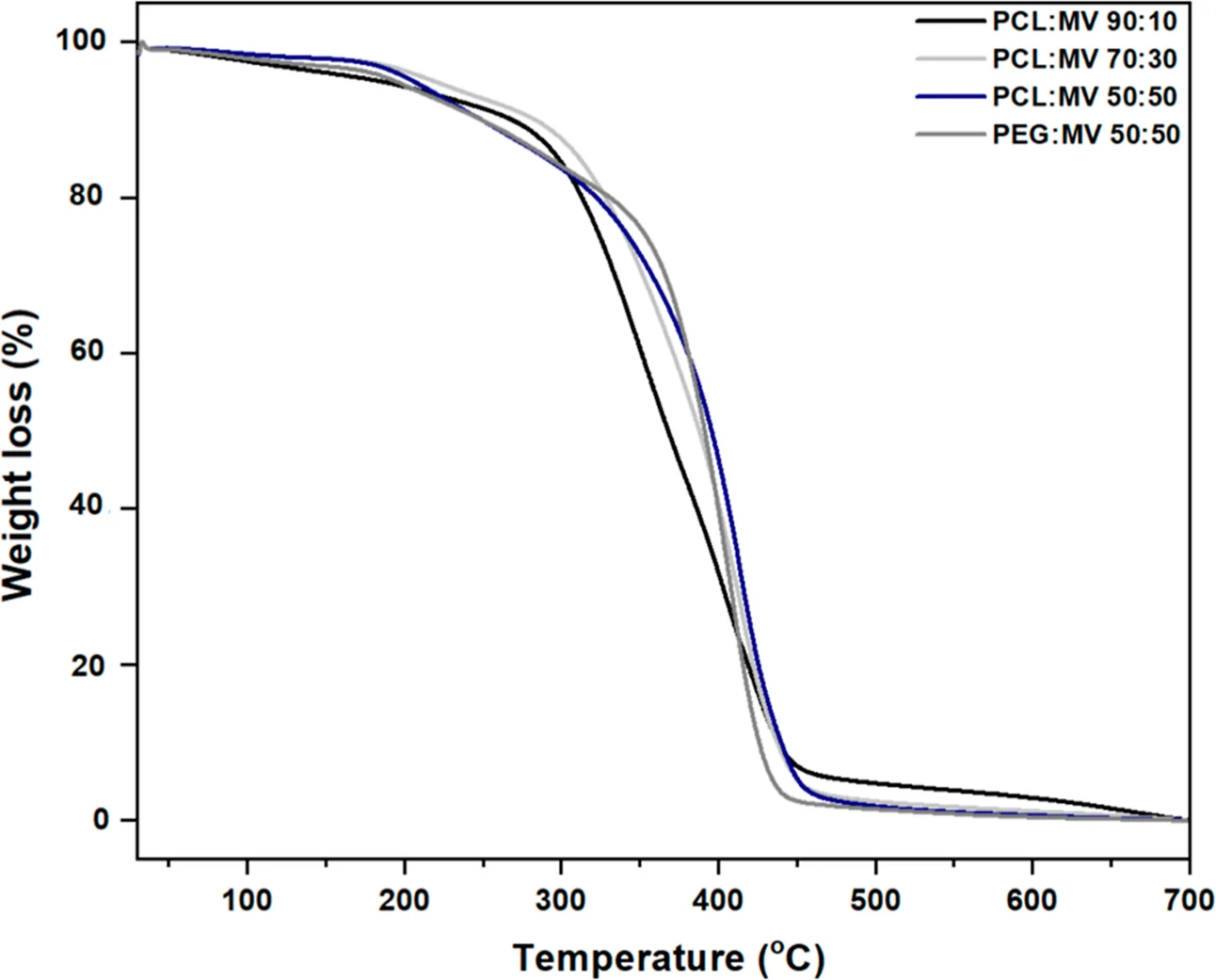
TGA curves of the four resin formulations showing their comparative thermal stability.

#### 3.3.3 Mechanical Performance

The mechanical behavior of the photocured networks is presented in Figure 5. The elastic modulus increased significantly with increasing methacrylated vanillin content in the PCL-based formulations, indicating the formation of progressively stiffer and more densely cross-linked networks. The PCL/MV 90:10 resin exhibited the lowest modulus, whereas the 50:50 formulation showed a substantial increase, confirming the reinforcing contribution of the aromatic MV component. The PEG/MV 50:50 formulation displayed the highest modulus among all systems, attributed to its high methacrylate functionality and the relatively rigid network structure formed by PEGDMA cross-linking.

**Figure 5:**
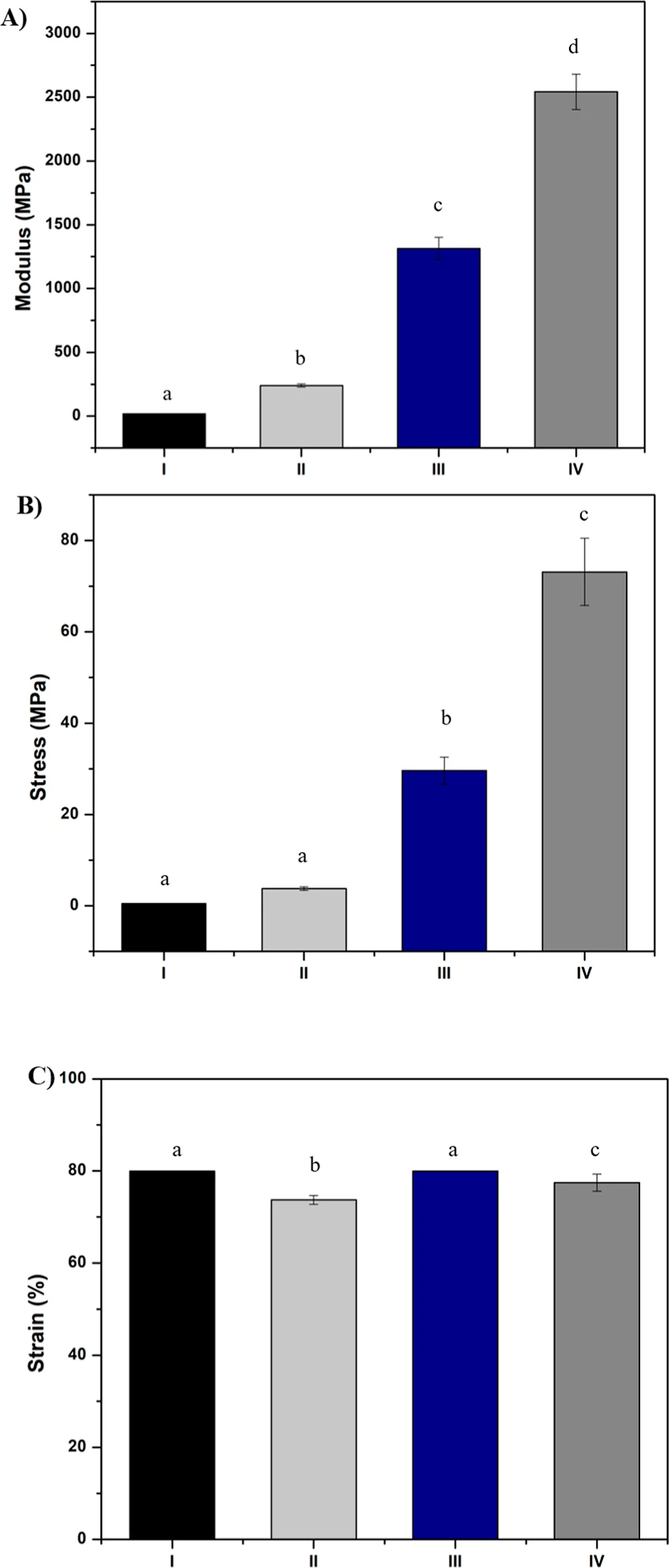
Mechanical properties of the photocured formulations (*n* = 3), showing (A) elastic modulus, (B) compressive stress, and (C) strain at break (%) for (i) PCL/MV 90:10, (ii) PCL/MV 70:30, (iii) PCL/MV 50:50, and (iv) PEG/MV 50:50. Different letters above the bars indicate statistically significant differences between formulations (*p* < 0.05), as determined by one-way ANOVA followed by Duncan’s multiple range test. Bars sharing the same letter are not significantly different.

In addition, a similar compositional trend was observed for the compressive stress at break. Increasing the MV fraction in the PCL/MV systems resulted in a stepwise increase in compressive stress, demonstrating improved load-bearing capability with higher cross-link density. The PEG/MV 50:50 resin again exhibited the highest compressive stress, consistent with its superior stiffness and structural robustness. Interestingly, despite statistically significant differences among some formulations, the strain at break remained within a relatively narrow range of approximately 70–80%, indicating that the networks generally retained appreciable ductility. Overall, the incorporation of methacrylated vanillin enhanced stiffness and compressive performance without severely compromising extensibility, while the PEG/MV system achieved the highest mechanical performance while retaining substantial deformation capability.

#### 3.3.4 Morphological Assessment

SEM analysis revealed apparent compositional effects on the microstructure of the photocured networks (Figure 6). In the top-view images, the PCL/MV 90:10 specimen exhibited a relatively smooth surface with fine cracks and isolated defects. As the MV fraction increased to 70:30 and 50:50, the surface became progressively rougher and more textured, consistent with the formation of a denser and more highly cross-linked network. In contrast, the PEG/MV 50:50 specimen showed a more homogeneous top-surface morphology, characterized by fused domains and smoother regions, compared with the PCL-based systems. Indeed, cross-sectional SEM images supported these observations. The PCL/MV 90:10 formulation displayed a relatively uniform interior with limited microstructural features, whereas increasing MV content produced noticeably rougher fracture surfaces and more complex internal morphology, particularly at the 50:50 composition. Such features indicate increased network rigidity and reduced plastic deformation during fracture. By comparison, the PEG/MV 50:50 network exhibited a dense and continuous morphology with well-developed structural features, reflecting its highly cross-linked character.

**Figure 6:**
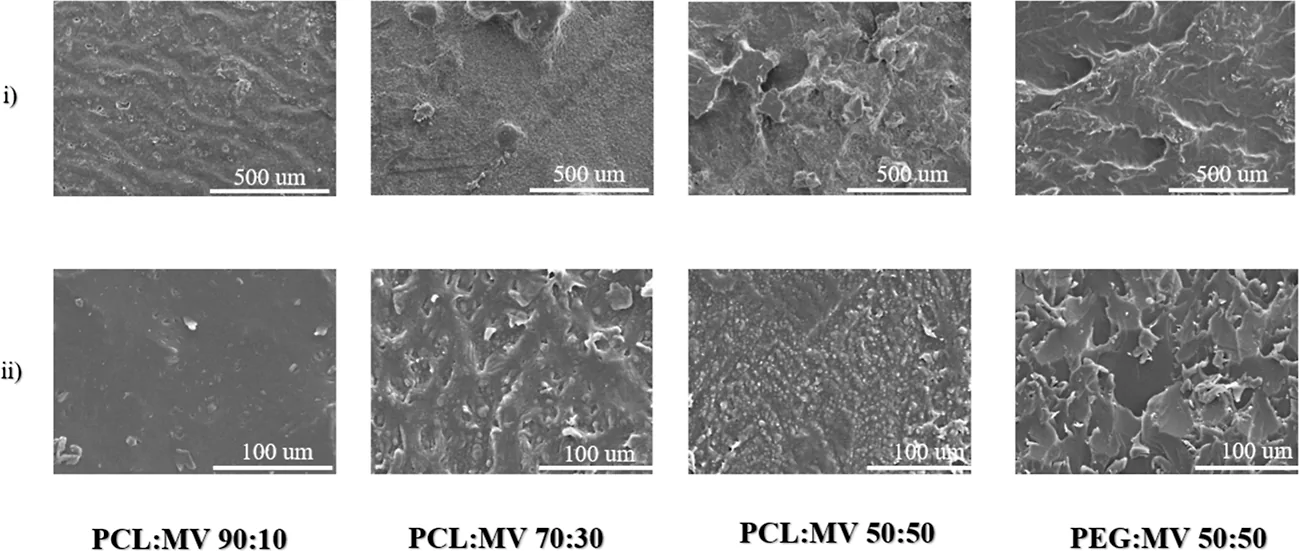
Scanning electron micrographs of the photocured specimens showing (i) top-view surface morphology and (ii) fracture cross-sectional morphology.

## 4 Conclusions

Photocurable hybrid resins based on methacrylated vanillin (MV) and glycidyl-methacrylate-functionalized poly­(ε-caprolactone) (PCL–GMA) were successfully developed for light-assisted 3D printing. FTIR and ^1^H NMR confirmed selective methacrylation of vanillin and methacrylate end-functionalization of the PCL macromer. Increasing the MV content significantly increased the curing depth within the PCL-based formulations, while the PCL/MV 50:50 and PEG/MV 50:50 formulations exhibited comparable curing depths. Increasing MV content also reduced resin viscosity, while all formulations remained readily printable. Printed specimens exhibited high dimensional fidelity, with only minor deviations from the CAD model after postcuring. The PCL/MV 50:50 resin provided a favorable balance between dimensional accuracy and high MV content and was therefore selected for the fabrication of porous lattice structures, which also showed low geometric error. Thermal analysis indicated that the main improvement in stability occurred between 10% and 30% MV, with limited further improvement at higher MV content. In addition, mechanical testing revealed a strong composition–property relationship: both the elastic modulus and compressive stress increased with the MV fraction, whereas the strain at break remained similar across all formulations, indicating retention of ductility. The PEG/MV 50:50 network displayed the highest stiffness, consistent with its highly cross-linked SEM morphology. Taken together, methacrylated vanillin proved to be an effective bioaromatic cross-linking unit for tuning structural, thermal, and mechanical behavior without compromising print quality. These findings demonstrate that simple formulation control enables precise tailoring of network properties, supporting the use of these hybrid resins for advanced light-assisted 3D printing of functional polymer components.
